# Does Size Matter? Testicular Volume and Its Predictive Ability of Sperm Production in Rams

**DOI:** 10.3390/ani13203204

**Published:** 2023-10-13

**Authors:** Rafael Montes-Garrido, Luis Anel-Lopez, Marta F. Riesco, Marta Neila-Montero, Cristina Palacin-Martinez, Cristina Soriano-Úbeda, Juan Carlos Boixo, Paulino de Paz, Luis Anel, Mercedes Alvarez

**Affiliations:** 1 ITRAULE, Animal Reproduction and Obstetrics, Department of Veterinary Medicine, Surgery, and Anatomy, University of León, 24071 León, Spain; rmong@unileon.es (R.M.-G.); mneim@unileon.es (M.N.-M.); cpalm@unileon.es (C.P.-M.); c.soriano.ubeda@unileon.es (C.S.-Ú.); jboixp00@estudiantes.unileon.es (J.C.B.); laner@unileon.es (L.A.); mmalvg@unileon.es (M.A.); 2 ITRAULE, Anatomy, Department of Veterinary Medicine, Surgery, and Anatomy, University of León, 24071 León, Spain; 3 ITRAULE, Cellular Biology, Department of Molecular Biology, University of León, 24071 León, Spain; mferrs@unileon.es (M.F.R.); ppazc@unileon.es (P.d.P.)

**Keywords:** caliper, ultrasonography, breeding soundness examination, reproductive performance, artificial insemination

## Abstract

**Simple Summary:**

Establishing the most suitable tool and the most accurate testicular volume determination formula could be essential in sperm production prediction. Traditionally, the caliper has been used to perform testicular measurements. In recent years, B-mode ultrasound has also been used as a measurement tool, providing, in addition to greater reproducibility as demonstrated in this work, other advantages such as being a non-invasive and non-ionizing technique that allows for the examination of the organ to see pathological processes. In the ovine species, the demand for seminal doses varies according to the genetic value of the donor males. The frequency of semen collection is high in males of great demand and genetic value. Testicular volume has a different influence on sperm production depending on the frequency of semen collection and the season of the year. This study demonstrated the effect of these factors on donor males to improve their reproductive performance.

**Abstract:**

Over the years, testicular volume has been used to evaluate the reproductive capacity of rams and the effects of different factors related to reproductive performance. The aim of this study was to determine the most suitable tool and formula to calculate testicular volume under field conditions to guarantee a more accurate determination of sperm production. First, testicles from 25 rams (*n* = 50) were measured in vivo and postmortem using calipers and ultrasonography during the breeding season (BS). The accurate testicular volume (ATV) was calculated through water displacement. In addition, the sexual status of donor rams was evaluated during a period of four years in a reproduction center, and the three most crucial groups in terms of genetic value and seminal collections were studied in the second part of this experiment: ER-NBS (Elite rams during the non-breeding season), ER-BS-S (Elite rams with a standard frequency of seminal collection), and ER-BS-O (Elite rams with a high frequency of seminal collection). The total testicular volume (TTV), testosterone (T), and total spermatozoa obtained from two consecutive ejaculates in the same day (SPERM) were measured, and the relationship between SPERM and TTV and T was analyzed to predict SPERM. Although all published formulas revealed statistically significant differences (*p* ≤ 0.05) from the ATV, our proposed formula (ItraULE) (Testicular volume = L × W × D × 0.61) did not show significant differences. In the second part of the study, in the ER as a model donor ram for its high genetic value and high demand from farmers, TTV and T showed strong positive correlations with SPERM (r = 0.587, *p* = 0.007 NBS; r = 0.684, *p* = 0.001 BS-S; r = 0.773, *p* < 0.0001 BS-O). Moreover, formulas were established to predict SPERM in these practical scenarios. In conclusion, the use of ultrasonography and a new formula adapted to rams could improve the prediction of SPERM considering crucial factors such as season and semen collection frequency.

## 1. Introduction

As it is well known, fifty percent of the reproductive potential of a flock is provided by the ram [1], affecting the profitability [2]. Traditionally, the size of the testes has been a variable to be taken into account in determining rams’ reproductive capacity [3]. Thus, breeding soundness evaluation (BSE) includes, among other factors, testicular measurements [4]. Several studies have evaluated the importance of testicular volume (TV) and weight on seminal quantity, quality [5,6], and fertility in ovine species [3]. Hence, TV has been key for estimating daily sperm output (DSO) in different species like stallions [7], rams [8], and bulls [9]. Spermatic efficiency (estimated DSO/real DSO) has been traditionally employed as a diagnostic criterion in testicular disorders [7], and TV is essential for DSO determination. Predicted DSO could be calculated with the general formula for several species DSO: 0.024 × total testicular volume (TTV)–0.76) [7]. Although general, the use of this formula is not widespread and standardized yet, which means that DSO is frequently not comparable within laboratories, and its analysis is not robust enough as an indicative factor of the ram reproductive performance.

Currently, different tools can be used to calculate TV: the classical caliper, which has been used in different species like bulls [10], dogs [11], and rams [12]; and the relatively recently incorporated ultrasonography, which has been used in equine [7,13], dogs [14], goats [15], rams [16], and humans [17]. Other tools such as the orchidometers, previously described by Prader [18] and Rochester [19], have had only a reduced use in human species and have not been used in ovine species. Therefore, various formulas have been used for calculating TV in different species. The formula for an ellipsoid (TV = length × height × width × 0.523) is the most commonly applied [11], being used in rams [16], dogs [20,21,22], and stallions [7,13]. The formula for a prolate spheroid (TV = length × width^2^ × 0.523) has also been used in bulls [9,23] and rams [24]. The empiric formula proposed by Lambert (TV = length × height × width × 0.71) [25] has been used in rams [26], dogs [14,27], goats [15], humans [28,29], and bucks [30]. Other formulas have been described for rams by different authors: TV = 0.0396 × length × (scrotal circumference)^2^ [31], TV = 1/6 × π × length × width^2^ × 0.945 [32,33], and TV = 2 × ((width/2)^2^ × π × length) [34]. However, it is not clear which measurements should be used to calculate TV, and no consensus has been reached on the best formula for rams. Because of this, we hypothesize that there is a possible misuse of accurate tools and methods for calculating TV.

In relation to the DSO and considering the working methods of ram semen collection, testicular density is necessary to determinate the sperm production under a certain frequency of semen collection. The testicular density is 1.038 g/mL in humans [35] and 1.04 g/mL in dogs and calves [36]. The daily sperm production per gram of testicular parenchyma is 21 to 25 × 10^6^ sperm in rams [37]. The seminiferous tubules containing mainly early sperm cells occupy 70–80% of the rams’ testes. However, the testes also contain interstitial tissue, including abundant testosterone-producing Leydig cells [38]. Therefore, changes in TV may reflect variations in testosterone production and profound implications for spermatogenesis [39] and sperm production [40].

Thus, this study aimed to optimize the method for calculating TV by choosing the most accurate and reproducible tool, obtaining a formula for calculating TV. In a second step, we characterized the role of TV as a feasible tool to predict sperm production. Finally, we proposed adjusted formulas for the prediction of sperm production considering different factors that influence the relationship between TV and sperm production.

## 2. Materials and Methods

### 2.1. Animals

Eighty-five sexually mature (age range from two to seven years) Assaf rams were used in the current study. All the rams were previously examined, and they did not have any disease or pathology. Animals were housed in the Animal Selection and Reproduction Center of the Junta de Castilla y León (CENSYRA) (Villaquilambre, León, Spain) and in Sheep and Goat Genetic Selection and Improvement Center of Castilla y León (OVIGEN) (Villalazán, Zamora, Spain), where they were fed daily with 2.5 kg of unifeed, including barley straw and grain, beet molasses, alfalfa hay, corn grain, soybeans, and a vitamin-mineral complex. The animals were provided with water ad libitum. The animal manipulations were conducted in agreement with the European Union Council Guidelines (2010/63/EU), following Spanish regulations (RD/53/2013) for the use of laboratory animals. All experiments were also approved by the institutional Animal Care and Use Committee at the University of León (ÉTICA-ULE-050-2022).

### 2.2. Experimental Design

#### 2.2.1. Experiment 1: Determination of Testicular Volume and the Most Suitable Formula

A total of 50 testes of 25 sexually mature rams housed in CENSYRA were measured in vivo using two measurement tools (calipers and ultrasonography). The rams were transported to Morajelo Selección slaughterhouse (Arcenillas, Zamora, Spain) to obtain carcasses for human consumption, and the disposed testes with all testicular wrappers were collected by the Itra-ULE laboratory (Veterinary Hospital of University of León, León, Spain). Testicular wrappers and the pampiniform plexus were removed. Testes were measured once again using calipers and ultrasound, and the accurate testicular volume (ATV) was calculated through water displacement. After all of these processes, a detailed study of the different formulas described in the literature [F1, TV = L × W × D × 0.523; F2, TV = L × W^2^ × 0.523; F3, TV = L × W × D × 0.71; F4, TV = 1/6 × π × W^2^ × L × 0.945; F5, TV = 0.0396 × L × (scrotal circumference)2; F6, TV = 2 × ((W/2)^2^ × π × L); F7, TV = (π × (W/2)^2^ × L) + (4/3 × π × (W/2)^3^] and their relation to the ATV was carried out. In addition, a study was performed using some formulas developed by our research group (Itra-ULE) [Fα, TV = −439.11 +29.767 × L + 26.266 × W + 35.956 × D; F8, TV = L × W × D × 0.61; F9, TV = (L × W × D × 0.523) + (2 × W); F10, TV = (L × W × D × 0.523) + (3 × W)], seeking a higher accuracy of the formula for the TV of the ram testes and maximizing the adjustment with respect to the ATV.

#### 2.2.2. Experiment 2: Analysis of the Sperm Production under Different Field Conditions

The following experiment was composed of two parts. In the first part of the survey, a retrospective study was conducted on the frequency of semen collection from donor males from 2018 to 2021 to find out the importance of this factor in a reproduction center. In the first step, ejaculates per month were calculated during the non-breeding season (NBS) and breeding season (BS) in all of the donor rams housed in the breeding center in the aforementioned years. In the second step, these rams were divided in two groups in both seasons (NBS and BS) considering the demand of their doses and their genetic value: elite rams (ERs) including males with high genetic value and high demand for seminal doses following the ASSAF.E criteria, and progeny testing rams (PTR) including the rest of the males available in the reproduction center. Based on this, the experimental groups in each season were as follows: PTR-NBS, PTR-BS, ER-NBS, and ER-BS. As the last step in this part of the experiment, the ER-BS were divided into two groups according to their frequency of seminal collection, considering the cut-off points established in a previous work published by our research group [41]: S (elite rams with a standard semen collection frequency) and O (overworked elite rams with an intensive semen collection frequency). In these animals, monthly ejaculates per male, doses per male, and ejaculates per male were compared.

In the light of the results obtained, the second part of the experiment consisted of a detailed analysis of the ERs in the reproduction center since they have the highest genetic value and were the most demanded. For this purpose, 60 sexually mature (age range from two to seven years) Assaf rams were used (20 males in each experimental group). These animals were used in the same way as the previous ER-NBS, ER-BS-S, and ER-BS-O experimental groups. Total testicular volume (TTV), serum testosterone (T), and total spermatozoa obtained from two consecutive ejaculates in the same day (SPERM) were evaluated after 1 month on the same seminal collection frequency. Ejaculates were collected by artificial vagina at 40 °C (IMV Technologies, L’Aigle, France), and a female was used as a decoy. The volume of ejaculates was measured by collecting them in Falcon^®^ type graduated sperm collection tubes. Sperm concentration was evaluated by an automatic cell counter (NucleoCounter SP-100, ChemoMetec, Allerod, Denmark). Semen samples fulfilled the criteria for semen doses preparation (ejaculate volume > 0.5 mL, sperm concentration > 3000 × 10^6^ sperm/mL, mass motility > 3) as previously described by Neila-Montero et al. [42], or they were discarded. Semen collections were carried out by the same investigator. To complete this second part of the experiment, correlations between SPERM and TTV and T were established in order to determine which physiological parameter could be used to predict how many spermatozoa a male would produce in each practical scenario. Lastly, linear regressions were established between the physiological parameters (TTV or T) that correlated in each experimental group (NBS, BS-S or BS-O) with respect to SPERM.

### 2.3. Clinical Examination and Testicular Size Determination by Calipers

**Figure 1:**
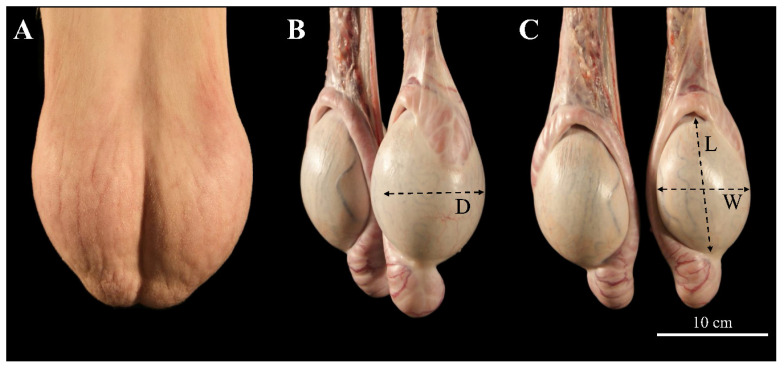
Determination of the testicular size in rams. (**A**) Caudo-cranial, (**B**) caudo-lateral, and (**C**) caudo-cranial planes. Anatomical structures: scrotal bag, testicles, epididimydes, and spermatic cord with testicular artery, pampiniform plexus, and ductus deferens. All testicular measurements were taken at the widest region of each axis: D (depth), W (width), and L (length) of caudo-cranial, lateral-lateral, and ventro-dorsal axis, respectively.

Every single male underwent a general clinical examination and visual inspection of the scrotum before being included in the study. Moreover, testicular palpation for the evaluation of consistency, symmetry, mobility, and sensitivity of the testes was performed (Figure 1A). In addition, epididymides and the pampiniform plexus were palpated to ensure that no observable gross pathology was present on the external genitalia. Then, a containment rack was used to restrain the rams in the standing position. The wool on both sides of the scrotum was shaved. Measurements of testicular depth (D, Figure 1B), width (W, Figure 1C), and length (L, Figure 1C) in cm were obtained using the calipers, and scrotal thickness was deducted. The measures were performed in triplicate. The measures were repeated when all testicular wrappers were removed after slaughtering the animals.

### 2.4. Ultrasound Examination

**Figure 2:**
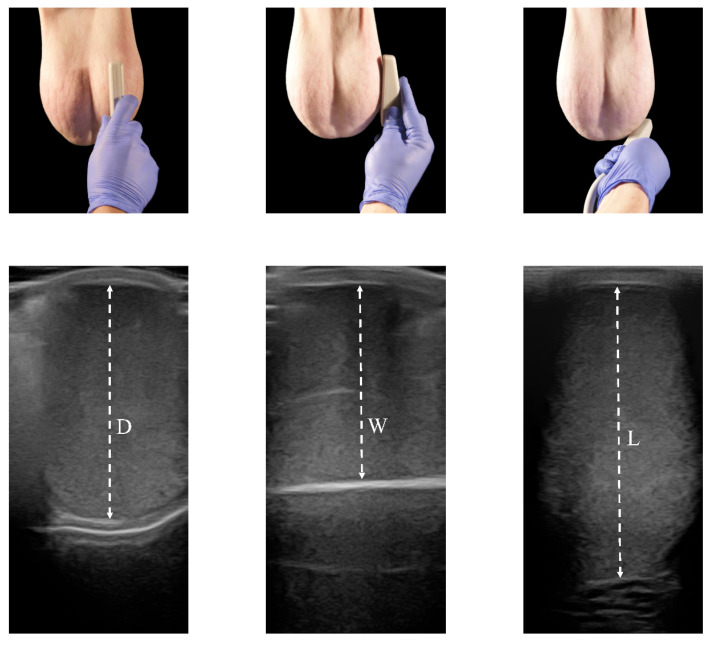
Ultrasound measurements. Placement of the probe for the measurement of the different testicular proportions. All testicular measurements were taken at the widest region of each axis: D (depth), W (width), and L (length) of caudo-cranial, lateral-lateral, and ventro-dorsal axes, respectively. The electronic caliper was positioned in the tunica albuginea at each end to count only the testicular parenchyma. All ultrasound images were viewed with the ultrasound beam emerging from the top region of the image.

All ultrasound measurements were carried out by the same investigator. All examinations were performed using a real-time ultrasound machine, EXAPAD (IMV, France), equipped with a 7.5 MHz linear array transducer attached directly to the skin. To eliminate the presence of air spaces, the transducer was covered with an abundant gel to facilitate ultrasound imaging. The scan was performed without pressure to avoid distorting the testicular shape. Images were obtained from the caudocranial (depth, D), lateral-lateral (width, W), and ventrodorsal (length, L) axes of the testes. Testicular D, W, and L were measured with electronic calipers integrated into the ultrasound machine. The cursors were fixed on the edges of the tunica albuginea. As for the calipers, testicular measurements were estimated in triplicate for each testicle. The echogenicity, homogeneity, and surface of the scrotal contents were also validated so that the animals used were considered to be within physiological standards. The measures were repeated when all testicular wrappers were removed after slaughtering the animals (Figure 2).

### 2.5. Accurate Testicular Volume (ATV) Determination

After measurement using both methods (calipers and ultrasound) and the slaughter of the rams, the epididymides and the pampiniform plexus were removed from the testes. Therefore, the ATV was calculated through water displacement according to Archimedes’ principle. The testicle was introduced in a container, and water displacement was quantified in a measuring cylinder [43].

### 2.6. Blood Obtention and Determination of Serum Testosterone

After all in vivo measurements, a programmed sanitary action was carried out at the reproduction center, and a blood sample was provided by them for testosterone analysis. The blood samples were collected from the jugular vein into a vacutainer tube without anticoagulants. The samples were refrigerated at 5 °C, and the blood serum was collected and stored at −20 °C until analysis. Serum testosterone was determined using the commercial ELISA kit L2KTW2 Immulite^®^ 2000 Total Testosterone by the Immulite 2000 XPi Immunoassay System (Siemens, Eschborn, Germany). According to the manufacturer’s instructions, the sensitivity was 0.15 ng/mL, and the intra- and inter-assay coefficients of variation were 5.1 and 7.2% when the average samples were 7.14 ng/mL.

### 2.7. Statistical Analyses

Data were analyzed with SAS/STAT™ version 9.1 statistical package (SAS Institute Inc., Cary, NC, USA) using a linear mixed-effects model (MIXED procedure), considering the male effect as a random factor. Results were displayed as the mean ± standard error of the mean (SEM). Mean values were considered statistically significant at *p* ≤ 0.05. Relation between ATV and TV estimated using different formulas was studied using Pearson’s correlation (CORR procedure). Linear regressions were used to establish the formulas to predict sperm production in the scenarios proposed in this work.

## 3. Results

### 3.1. Reproducibility of the Measuring Tool

**Figure 3:**
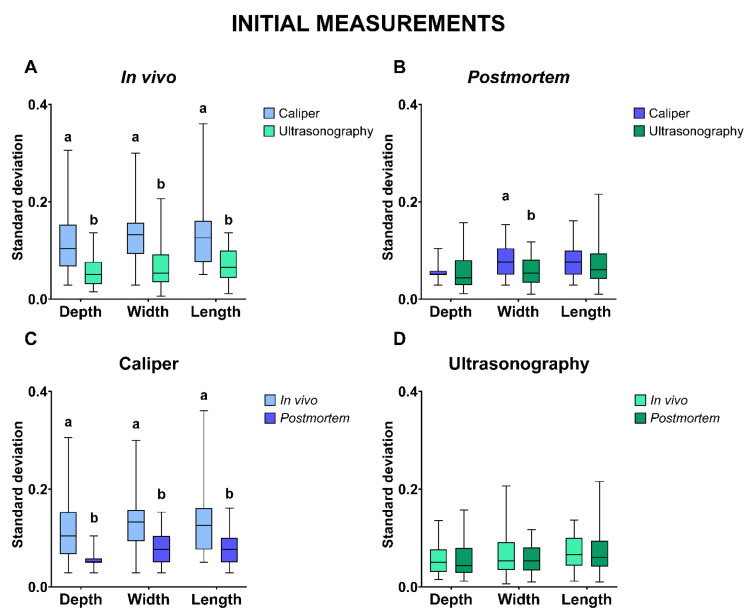
Standard deviation comparisons of initial measurements of morphometric testicular evaluation. Standard deviation comparisons of in vivo (**A**) and postmortem (**B**) measurements of testicular depth, width, and length between calipers and ultrasonography. Standard deviation comparisons of measurements using caliper (**C**) and using ultrasonography (**D**) of testicular depth, width, and length between in vivo and postmortem. The same 50 testicles from 25 male were compared. Different lowercase superscript letters (a, b) indicate differences (*p* ≤ 0.05) between groups within the same measurement.

Firstly, to estimate the reproducibility of the tool, the standard deviations (SD) of testicular D, W, and L determined with both calipers and ultrasonography were calculated in vivo and postmortem. The SD of D, W, and L was significantly higher using the caliper than the ultrasound for in vivo measurements (D: 0.111 ± 0.008 vs. 0.059 ± 0.005; W: 0.130 ± 0.008 vs. 0.068 ± 0.007; L: 0.132 ± 0.009 vs. 0.070 ± 0.005; *p* ≤ 0.05; Figure 3A). According to postmortem measurements, the standard deviations of the testicular width measured using calipers were significantly higher (*p* ≤ 0.05) than measures made using ultrasonography (Figure 3B). Concerning caliper measurements, the standard deviations of all in vivo measurements were significantly higher (*p* ≤ 0.05) than all postmortem measurements (Figure 3C). By contrast, the in vivo and postmortem measurements made using ultrasonography did not differ (*p* > 0.05; Figure 3D).

### 3.2. Testicular Measurements and the Most Suitable Formula to Calculate Testicular Volume

**Figure 4:**
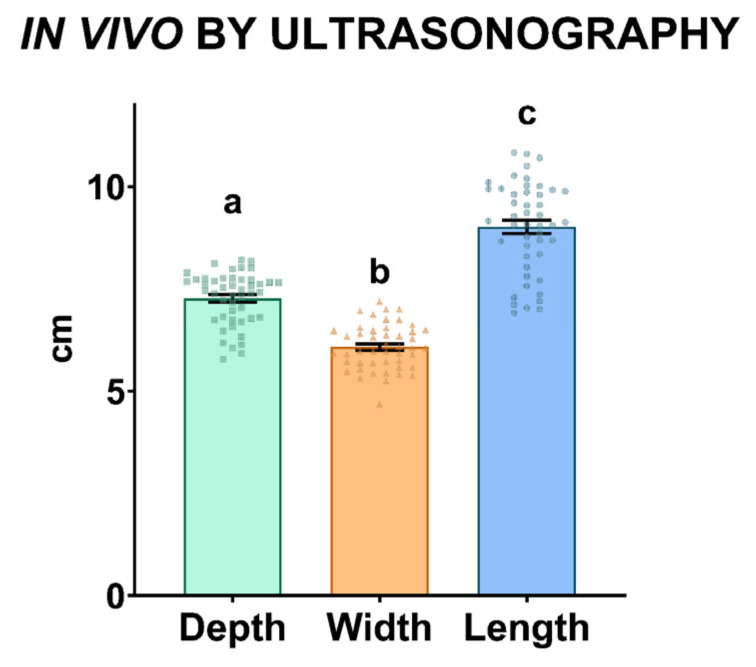
Comparison among the testicular depth, width, and length measurements performed using ultrasonography. The same 50 testicles from 25 male were used. Different lowercase superscript letters (a, b, c) indicate differences (*p* ≤ 0.05) among the different measurements.

In order to demonstrate the importance of including different measurements (three testicular dimensions) to calculate the TV, depth, width, and length were compared in 25 animals. All testicular measurements were significantly different (*p* ≤ 0.05) from each other (Figure 4) for in vivo evaluations.

**Figure 5:**
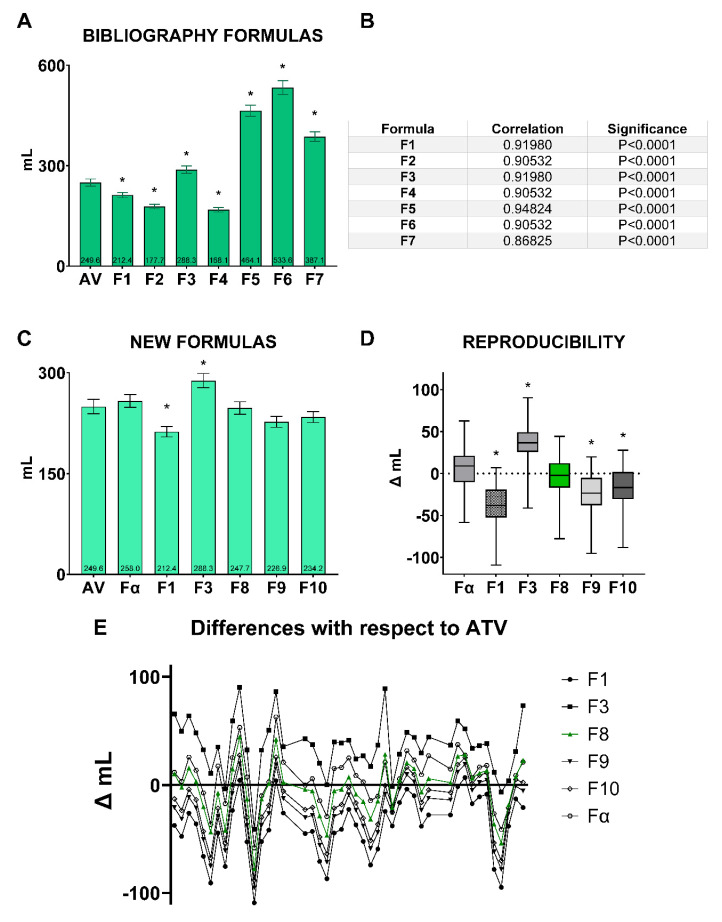
(**A**) Testicular volume (TV) calculated using the formulas described in the literature (F1 to F7) and their relation to the accurate testicular volume (**ATV**). (**B**) Correlation coefficients and significance between formulas F1 to F7 and ATV. (**C**) TV calculated with F1, F3, and the new formulas designed by our research group. (**D**) Variations between TV calculated with each formula and ATV. (**E**) Variations between TV calculated with each formula and ATV in each sampled testicle. The symbol “*” in superscript indicates differences (*p* ≤ 0.05) with respect to AV (**A**,**C**) and Fα (**D**). **Formula legend**: **ATV**, accurate testicular volume calculated through water displacement; **F1**, Testicular volume (TV) = L × W × D × 0.523; **F2**, TV = L × W^2^ × 0.523; **F3**, TV = L × W × D × 0.71; **F4**, TV = 1/6 × π × W^2^ × L × 0.945; **F5**, TV = 0.0396 × L × (scrotal circumference)^2^; **F6**, TV = 2 × ((W/2)^2^ × π × L); **F7**, TV = (π × (W/2)^2^ × L) + (4/3 × π × (W/2)^3^); **Fα**, TV = −439.11 +29.767 × L + 26.266 × W + 35.956 × D; **F8,** TV = L × W × D × 0.61; **F9**, TV = (L × W × D × 0.523) + (2 × W); **F10**, TV = (L × W × D × 0.523) + (3 × W).

On the other hand, several formulas have been studied, which are shown in Figure 5A,B. All formulas revealed statistically significant differences (*p* ≤ 0.05) from the ATV measured through water displacement (Figure 5A). Moreover, all formulas showed strong positive correlations with ATV (Figure 5B).

As there are statistically significant differences between the three testicular dimensions (depth, width, and length), and all published formulas revealed statistically significant differences (*p* ≤ 0.05) from the ATV measured through water displacement, we designed new formulas using all three testicular dimensions, and these formulas were calculated as follows: **F8**, TV = L × W × D × 0.61; **F9**, TV = (L × W × D × 0.523) + (2 × W); **F10**, TV = (L × W × D × 0.523) + (3 × W). Moreover, the formula obtained through a linear regression on our data was obtained and included in this study (**Fα**, TV = −439.11 +29.767 × L + 26.266 × W + 35.956 × D). Thus, the new formulas did not differ from the ATV (*p* > 0.05; Figure 5C). By contrast, all formulas, except **F8** (ItraULE TV = L × W × D × 0.61), showed significant differences (*p* ≤ 0.05) when we compared the variation between the TV calculated with each formula and the most accurate formula calculated using linear regression (Fα; Figure 5D). In addition, the individual variation between TV calculated with each formula and ATV in each sampled testicle is represented in Figure 5E.

### 3.3. Semen Collection and Ram Working Method in a Reproduction Center under Field Conditions

**Figure 6:**
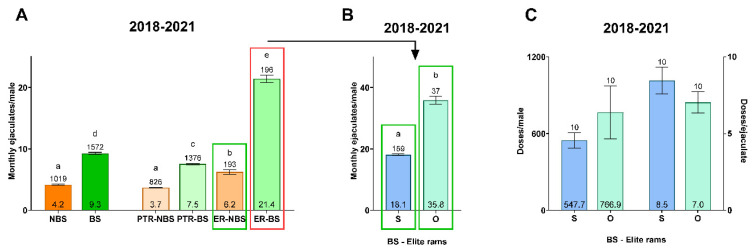
A retrospective view of field conditions in a reproduction center in terms of semen collection and working approach conditioned by the genetic status of the rams. (**A**) Mean ejaculates per month and ram in **NBS** (non-breeding season), **BS** (breeding season), **PTR-NBS** (progeny testing rams in NBS), **PTR-BS** (progeny testing rams in BS), **ER-NBS** (elite rams in non-breeding season), **ER-BS** (elite rams in breeding season). (**B**) Monthly semen collection rate from 2018 to 2021 in elite rams during BS classified in two seminal collection ratios: **S** (standard semen collection frequency like BS-S group) and **O** (overworked rams with a semen collection frequency like BS-O group). (**C**) A descriptive survey of the sexual work performance of the 10 most used males according to their genetic value. In all three graphs, the sample size is shown above the error bar; the mean per group is shown at the bottom of the bar. Different lowercase letters (a–e) indicate statistical differences (*p* ≤ 0.05) among different experimental groups.

In this second part of the work, a retrospective study was conducted over 4 years, showing how the rams were managed according to their genetic value and demand by the breeders of the selection scheme. Concerning season, the number of ejaculates per month was significantly higher in BS compared to NBS (9.3 ± 0.2 vs. 4.2 ± 0.1; *p* ≤ 0.05; Figure 6A). The retrospective study showed that the ER semen samples were significantly more demanded by farmers than PTR (21.4 ± 0.6 vs. 7.5 ± 0.1 in BS; 6.2 ± 0.4 vs. 3.7 ± 0.1 in NBS; *p* ≤ 0.05). Focusing on ERs, the field conditions in NBS were taken as a reference for the second part of the experiment developed for the estimation of SPERM in that season (see the next section). On the other hand, ERs utilized during BS were divided into two groups differentiated according to the monthly ratio of ejaculates per male (Figure 6B). Thus, overworked ERs showed a significantly higher number of monthly ejaculates compared to ERs with a standard work regime (35.8 ± 1.3 vs. 18.1 ± 0.3; *p* ≤ 0.05). Lastly, in a descriptive survey of the 10 most-used males from the reproduction center, it could be seen how the rams had a lower yield (Figure 6C).

### 3.4. Application of Testicular Volume and Serum Testosterone to Estimate Sperm Production

**Figure 7:**
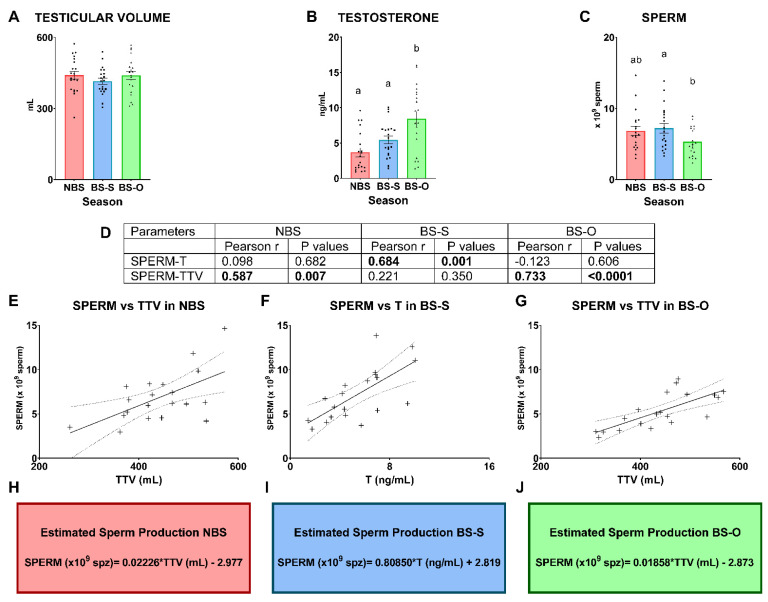
Results of the second part of the experiment. A total of 20 males were used in each experimental group according to the rate of seminal collections: **NBS**: males used in non-breeding season with a standard working rate of seminal collections in a reproduction center, **BS-S**: males used in breeding season with a standard seminal collection rate of 2 ejaculates per day/2 days per week, **BS-O**: males in breeding season with an overworked seminal collection rate of 2 ejaculates per day/5 days per week. (**A**) Testicular volume (mL); (**B**) Testosterone (ng/mL); (**C**) Sperm (×10^9^ sperm); In graphs (**A**–**C**), means (±SEM) are shown. Also, graph dots represent individual male values. Different lowercase superscript letters indicate differences (*p* ≤ 0.05) among the experimental groups. (**D**) Correlation coefficients between sperm and physiological parameters: total testicular volume (TTV) and testosterone (T); (**E**) Linear regression between TTV and Sperm in NBS. (**F**) Linear regression between testosterone (T) and sperm in BS-S. (**G**) Linear regression between TTV and Sperm in BS-O. On all graphs, the continuous lines represent the balance line. The dots lines mark the 95% confidence interval. The equation for each practical scenario is shown in the following boxes of the figure: (**H**) Equation for estimated sperm production in NBS; (**I**) Equation for estimated sperm production in BS-S; (**J**) Equation for estimated sperm production in BS-O.

The results of the second part of the experiment based on the retrospective study of the reproduction center under field conditions are shown in Figure 7. First, all semen samples met the minimum criteria established for the preparation of semen doses (ejaculate volume > 0.5ml, sperm concentration > 3000 × 10^6^ sperm/mL, mass motility > 3). According to the TV, non-significant differences were found among the experimental groups (440.4 ± 16.8 cm^3^ NBS; 414.9 ± 13.2 cm^3^ BS-S; 439.4 ± 17.6 cm^3^ BS-O; *p* > 0.05). The highest testosterone levels were observed in the rams of the BS-O group (8.4 ± 1.0 ng/mL; *p* ≤ 0.05). Concerning sperm production, BS-S rams had higher sperm production than BS-O rams (*p* ≤ 0.05), and NBS rams showed no significant differences (*p* > 0.05) with any of the other experimental groups (6.8 ± 0.6 × 10^9^ sperm NBS; 7.2 ± 0.7 × 10^9^ sperm BS-S; 5.3 ± 0.4 × 10^9^ sperm BS-O). Correlations between sperm production and T and TTV are shown in Figure 7D. SPERM showed a significant strong positive correlation with testosterone in the BS-S group (R^2^ = 0.684) (*p* ≤ 0.05). Also, SPERM correlated strongly and positively with TTV in the NBS and BS-O groups (R^2^ = 0.587 and R^2^ = 0.733, respectively) (*p* ≤ 0.05). Lastly, non-significant correlations were found in the rest of the relationships among SPERM, TTV, and T in BS and NBS (*p* > 0.05). On the other hand, the linear regression of SPERM versus TTV in NBS is shown in Figure 7E, and the equation for estimating sperm production under these conditions is SPERM (×10^9^ spz) = 0.02226 × TTV (mL) − 2.977 (shown in Figure 7H). Moreover, the linear regression of SPERM versus T in BS-S is shown in Figure 7F, and the equation for estimating sperm production under these conditions is SPERM (×10^9^ spz) = 0.80850 × T (ng/mL) + 2.819 (shown in Figure 7I). Finally, the linear regression of SPERM versus TTV in BS-O is shown in Figure 7G, and the equation for estimating sperm production under these conditions is SPERM (×10^9^ spz) = 0.01858 × TTV (mL) − 2.873 (shown in Figure 7J).

## 4. Discussion

Donor rams housed in reproduction centers are the main individuals on whom we focused our efforts in this study. It would be of great interest to know the production capacity of the rams in order to optimize the management of a reproduction center. To this end, a rapid and reliable measurement with a predictive ability of reproductive performance would be ideal, and the TV could be such a measure. In addition, a laboratory-measurable parameter with an impact on sperm production would also be of interest as a predictor of sperm production, and that parameter could be testosterone [5,44].

At first, regarding the tool to be used for TV determination, ultrasonography would be the most reliable and reproducible tool according to our results, with lower standard deviations in the measurements performed using the ultrasound technique compared to those performed using calipers. Pricking et al. [45] demonstrated significant differences between the testicular volume calculated by calipers and ultrasound in the equine species. In the same way, the results obtained by Prader et al. [46] also supported our data by presenting a significantly higher standard deviation in testicular volumes calculated with clinical methods compared to ultrasound methods in canine species. Although caliper measures are reliable, inexpensive, and simple [23], compared to the B-mode ultrasound technique, ultrasound provides many general advantages such as its non-invasiveness, non-ionizing property, non-damaging property, real-time visualization, sequential information, and storage for future assessment [47]. Therefore, the ultrasound technique is useful to interpret uncertain clinical findings, to detect early stages of pathological processes, to monitor changes in lesions, and to study the echogenicity of the parenchyma [16,41,47]. For all these reasons, the choice of ultrasound as the most accurate and reproducible tool has been endorsed in other species such as dogs [11,46], bulls [48], stallions [45], and humans [49]. Nowadays, ultrasound equipment with electronic calipers are very accessible to all veterinary technicians, and the determination of testicular measurements could be one more use of this equipment in daily clinical practice.

For the determination of TV in rams, several formulas have been used [16,24,26,31,32,33,34], but according to our results, none is accurate enough to calculate the TV in ovine species. In several studies, the formula used is not important because they are comparative studies such as a comparison over time considering seasonal variations [50], between different ages [16], or at different seminal collection frequencies [41]. Moreover, it has been demonstrated in our results and in other studies [45,46] that there are positive correlations between these formulas described in the literature and ATV (Figure 5B). However, we would like to know whether TV could be a key factor in the prediction of sperm production; therefore, improving the accuracy of its precise calculation would help us to achieve this goal. In addition, we would also like to know if the study of other factors, specifically serum testosterone, would be crucial to more precisely know the sperm production.

The formula developed by our research group (ItraULE formula: TV = L × W × D × 0.61) did not show significant differences, neither in global values (Figure 5C) nor in individual differences (Figure 5D) between the volume value obtained using the formula and the real postmortem value measured using water displacement. This indicates that it is more accurate in the different repetitions of TV measurements in the different individuals measured.

Once the measurement tool and the best formula for calculating TV were determined, these data were used for a practical application of TV under field conditions. For this, a study during the years of 2018–2021 of a reproduction center was conducted (Figure 6). The results obtained in this study showed that the reproductive potential of genetically valuable animals (elite rams) is under-optimized by the excessive regime of semen collection due to the high demand by farmers. The experimental conditions served as a model for our study to predict SPERM. The high demand of seminal doses from certain males makes the yields of these rams lower than they could produce with better-organized management. This fact has its repercussion in the seminal quality, as it has already been demonstrated in other works of our research group [41]. According to Montes-Garrido et al. [41], sperm motility and functionality were lower at 6 h post-collection (the time at which vaginal artificial insemination is performed) in the spermatozoa of rams that were overworked. Moreover, the commercial potential of overworked rams is also reduced for frozen-thawed doses, since previous authors have described the lower cryotolerance of spermatozoa produced in a high regime of collection due to a higher oxidative stress through the activity of glutathione peroxidase and superoxide dismutase [51]. Despite all this, it would be interesting to consider the economic impact of specific rams and the need to use them to accelerate genetic improvement. Therefore, overworked males were considered as a model for the second part of the experiment for SPERM prediction.

Considering that the seminiferous tubules occupy 67–83% of the rams’ testes and that most of the cells forming the seminiferous tubules are sperm cells in their different stages of development, from spermatogonia to immature spermatozoa [38], the main practical application of TV could be the prediction of sperm production. There is no consensus on the variation of sperm production throughout the year. On the one hand, Kafi et al. [52] demonstrated that total sperm output is constant throughout the year and that it was not affected by month. These results agree with those obtained by us in rams that were not overworked. On the other hand, several studies have shown significant differences in sperm production among the different months of the year [44,53]. However, all of the studies have proved that the testicular size varied significantly throughout the year, with the highest value being recorded in BS [50,52,54,55]. This justifies that it is not possible to establish a strong positive correlation between TV and sperm production that would be valid throughout the year.

In our study, a strong positive correlation was demonstrated in males with the semen collection work planned at a reproduction center in NBS (Figure 7D). In this season, we hypothesize that the TV depends directly on the number of sperm cells available to produce spermatozoa since the testosterone, which is other important factor that influences spermatogenesis, is at the lowest levels, as previously demonstrated by other authors [26,44,52]. Furthermore, based on this correlation, we developed a formula to predict sperm production under these conditions. By contrast, we found the opposite scenario in rams working at a standard semen collection rate in BS. A positive correlation between TV and sperm production was not demonstrated. However, a strong positive correlation between serum testosterone and sperm production was demonstrated (Figure 7D) in these conditions. Firstly, hormone production was shown to distinctly vary with melatonin secretion in the rams [56], and, specifically, melatonin increased testosterone production in the ram Leydig cells [57]. Moreover, melatonin promoted insulin-like growth factor-1 and decreased estrogen synthesis in Sertoli cells [58], which plays an important role in the regulation of testosterone production [59]. It is widely known that the seminal, blood, and pineal melatonin concentrations increase during the dark phase of the light–dark cycle [60], and light signal results in being anti-gonadotropic in short-day animals such as ovine species [61]. Thus, melatonin levels are maximal in terms of their nocturnal duration [62], and the hormones related to reproduction have their maximum values in BS, i.e., in short days [26]. Among these reproductive hormones, testosterone plays a key role in spermatogenesis. Although the classical view is that FSH stimulates spermatogenesis and LH stimulates testosterone production [63], spermatogenesis was qualitatively and quantitatively maintained by testosterone alone in rats immunized against GnRH [64], and this fact was also demonstrated in men with an inactivating mutation of the FSH-R gene [65] and in an FSH-β deficient mouse [66], in which FSH is not essential for male fertility. Moreover, another study determined that basal levels of sperm and germ cells production were maintained by intratesticular testosterone [67]. Under these conditions, the serum testosterone level may be the most suitable predictor of sperm production and not the testicular size. In this sense, considering that the daily sperm production per gram of testicular parenchyma is 21 to 25 × 10^6^ sperm [37,68] in BS, the sperm ejaculated by a male in commercial reproduction centers in a standard work routine for semen collection is less than the sperm the male produces physiologically [41], and testicular size could not be the most reliable parameter to predict sperm production.

In reproduction centers, due to the increasing demand for semen doses from males of a high genetic value, as demonstrated in this work, rams are overworked when producing spermatozoa, and the high frequency of semen collection has negative consequences on semen quantity and quality [41]. When rams are overworked, the doses obtained per ejaculate are lower. In this scenario, we demonstrated a strong positive correlation between sperm production and TV, and a formula to predict sperm production was estimated. We hypothesize that the amount of ejaculated spermatozoa could be practically the same as the spermatozoa produced in the gonads. These experimental conditions are close to those used in other species such as stallions [7,45,69,70] and jacks [71], where DSO is calculated after overworking the animals with several seminal collections; therefore, the sperm production depends solely on testicular size [45].

## 5. Conclusions

To summarize, improving the formula for calculating TV in rams (ItraULE formula: TV = L × W × D × 0.61) was possible using new technologies such as ultrasonography, which provided more reproducible measurements than the caliper. Testicular volume could be a good indicator of sperm production for all rams in NBS and in BS for overworked rams, since the size of the organ determines its production capacity. On the other hand, during BS, when males are in a standard sexual rhythm, testosterone could be the parameter that best predicts sperm production.

## Data Availability

The data presented in this study are available on request from the corresponding author.
